# NRIP/DCAF6 stabilizes the androgen receptor protein by displacing DDB2 from the CUL4A-DDB1 E3 ligase complex in prostate cancer

**DOI:** 10.18632/oncotarget.15308

**Published:** 2017-02-14

**Authors:** Hsin-Hsiung Chen, Ping Fan, Szu-Wei Chang, Yeou-Ping Tsao, Hsiang-Po Huang, Show-Li Chen

**Affiliations:** ^1^ Graduate Institute of Microbiology, College of Medicine, National Taiwan University, Taipei 100, Taiwan; ^2^ Department of Ophthalmology, Mackay Memorial Hospital, Taipei 104, Taiwan; ^3^ Graduate Institute of Medical Genetics and Proteomics, College of Medicine, National Taiwan University, Taipei 100, Taiwan

**Keywords:** NRIP/DCAF6, DDB2, AR, Cul4-DDB1, cribriform prostate cancer

## Abstract

Both nuclear receptor interaction protein (NRIP) and DNA damage binding protein 2 (DDB2) belong to the Cullin 4 (CUL4)-DDB1 binding protein family and are androgen receptor (AR)-interacting proteins. Here, we investigated the expression patterns of the NRIP, DDB2 and AR proteins in human prostate cancer tissues and found that the expression levels of NRIP and AR were higher, but the DDB2 level was lower, in prostate cancer tissues than in non-neoplastic controls, suggesting NRIP as a candidate tumor promoter and DDB2 as a tumor suppressor in prostate cancer. Furthermore, both NRIP and DDB2 shared the same AR binding domain; they were competitors for the AR, but not for DDB1 binding, in the AR-DDB2-DDB1-CUL4A complex. Conclusively, NRIP stabilizes the AR protein by displacing DDB2 from the AR-DDB2 complex. Consistent with our hypothesis, a specific expression pattern with high levels of NRIP and AR, together with a low level of DDB2, was found more frequently in the human prostate cancer tissues with a cribriform pattern than in non-cribriform tumors, suggesting that disruption of the balance between NRIP and DDB2 may change AR protein homeostasis and contribute to pathogenesis in certain aggressive types of prostate cancer.

## INTRODUCTION

Previously, we determined that the nuclear receptor interaction protein (NRIP; also named DCAF6 and IQWD1) is a transcriptional cofactor that enhances androgen receptor (AR)-mediated transcriptional activity [1] and an AR-targeted gene. In addition, NRIP can protect the AR protein from proteasome degradation, although the mechanism is unclear [2]. NRIP also has been reported to be a member of the DDB1 and Cullin 4 (CUL4)-associated factors (DCAF) family [3]. The NRIP protein is composed of 860 amino acids and contains seven WD-40 repeats and one IQWD1 [1]. Moreover, we also found that NRIP is a human papillomavirus 16 E2-interacting protein and acts as a scaffold to recruit E2 and calcium/calmodulin to prevent polyubiquitination and degradation of E2, resulting in enhanced E2 stability [4]. NRIP is associated with human diseases, high expression levels of IQWD1 (NRIP) in breast cancer tissues are significantly associated with adverse clinical outcomes [5] and NRIP expression is found in six human malignancies (esophageal, colon, breast, ovarian, skin and pancreatic cancers) [6]. *NRIP* is also one of the candidate genes involved in cerebral visual impairment, which is causally related to variants of one or multiple genes, including *NRIP*, with an autosomal recessive transmission pattern [7].

DNA damage binding protein 2 (DDB2, also named p48 and XPE), like NRIP, belongs to the DCAF family [8]. Recent reports indicate that the DDB2–DDB1–CUL4 complex is involved in the nucleotide excision DNA repair (NER) pathway response to UV irradiation [9] and protein degradation control [10, 11]. NormalDDB2 is an important component of the NER pathway that, when impaired, may cause xeroderma pigmentosum [12], human skin cancer [13, 14], and tobacco-related lung cancer [15]. In addition, DDB2 inhibits tumor growth by various mechanisms, such as limiting the cancer stem cells in ovarian cancer [16, 17]. The DDB2 protein level is reduced in high-grade colon cancers [18]. DDB2 can suppress the invasion of breast cancer by decreasing NF-κB activity [19, 20] and indirectly suppresses ovarian cancer proliferation [21]. Hence, DDB2 also acts as a tumor suppressor. In terms of DDB2 functions in ubiquitination and degradation, a current report revealed that DDB2 could facilitate tumorigenesis of gastric cancer via ubiquitination and degradation of the tumor suppressor progestin and adipoQ receptor family member 3 [22]. Similarly, we also found that DDB2 mediates ubiquitination and degradation of the AR via the CUL4A-DDB1 E3 ligase complex [23].

The AR is causally linked to prostate cancer and androgen-AR signaling is critical for prostate cancer development and progression [24, 25]. In general, androgen binds to the AR; the bound AR molecules become homodimers and translocate into the nucleus, binding to the cognate DNA response elements. Coregulators (coactivators and corepressors) are then recruited to increase or decrease the signals of the hormones to the transcriptional machinery and result in the initiation and progression of prostate cancer [26]. Therefore, most prostate cancers can be treated initially by androgen deprivation. However, due to increased protein levels of AR or AR mutations that cause AR-resistance to anti-androgen treatment, patients may relapse, with the development of a castration-therapy-resistant stage of prostate cancer [27]. Consequently, it would be interesting to investigate the correlation between AR-interacting proteins and prostate cancer. Previously, we found that both NRIP and DDB2 are AR-interacting proteins [23], and belong to the DCAF-associated protein family. Therefore, we were interested in examining the NRIP, DDB2 and AR protein expression profiles in human prostate tumors with the aim of elucidating the interaction mechanism of these three proteins and their roles in prostate cancer. Here, we showed that NRIP, like DDB2, was a DCAF-associated protein and could form a CUL4A-DDB1 complex. NRIP and DDB2 bound to the same domain of the AR protein. Hence, NRIP attenuated the association between DDB2 and the AR and an NRIP mutant that lacks DDB1 binding ability was still capable of interfering with the interaction between DDB2 and AR, indicating that NRIP and DDB2 are competitors for the AR, but not for DDB1 binding, in the AR-DDB2-DDB1-CUL4A complex. In clinical significance, NRIP was a candidate tumor promoter and DDB2 was a tumor suppressor in prostate cancer. The relationship between the expression of NRIP, AR and DDB2 and the pathological sub-types of prostate cancer, we found that a specific pattern of high expression of NRIP and AR and simultaneous low expression of DDB2 was detected more frequently in cribriform tumors than in non-cribriform tumors.

## RESULTS

### NRIP is highly expressed in prostate tumors

**Figure 1:**
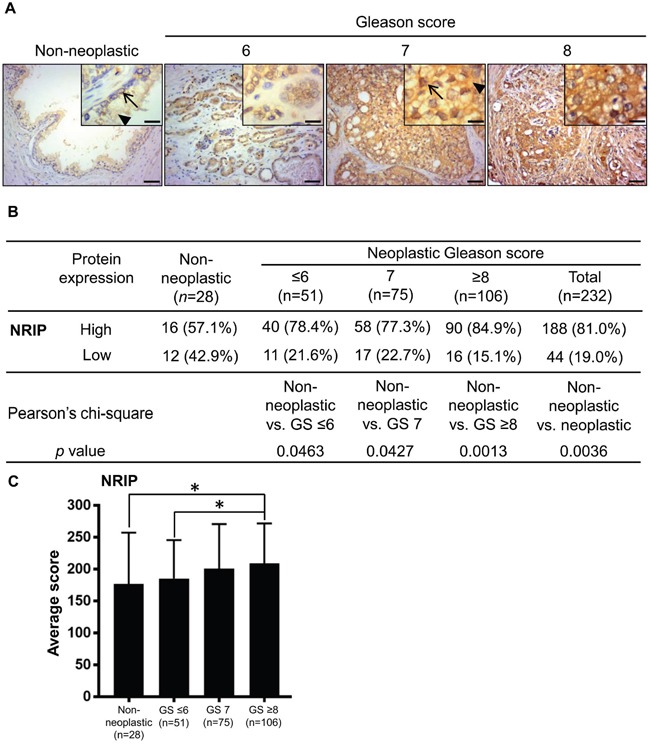
NRIP expression is increased in human prostate cancer tissues compared to non-neoplastic prostate tissues **A**. Representative images of immunohistochemistry staining for NRIP expression in non-neoplastic prostate tissues (n = 28) and prostate cancer tissues (n = 232), including Gleason Score ≤ 6 (n = 51), Gleason Score = 7 (n = 75), and Gleason Score ≥ 8 (n = 106). Left to right represent non-neoplastic, Gleason Score ≤ 6, Gleason Score = 7, and Gleason Score ≥ 8 groups. Brown: NRIP. Blue: hematoxylin counterstain. Upper right insets: magnified figures, Scale bar = 12.5 μm for insets, 50 μm for others. Arrow: NRIP expression in nucleus; arrowhead: cytosol expression. **B**. Comparison of the NRIP expression levels in non-neoplastic tissues and tumors of different grades. The intensity of NRIP expression was scored as follows: 0 = negative staining, 1 = weak brown staining, 2 = intermediate brown staining, and 3 = dark brown staining. The extent was scored as the percentage (scored as 0-100%) of the positively-stained area. The total score was the product of the intensity and extent scores, from 0 to 300. The score equal to or greater than 150 was defined as “high” expression and otherwise as “low” expression. Statistical analysis was performed using Pearson's chi-square test. GS, Gleason Score. **C**. The NRIP expression level, based on its average immunohistochemistry score, was significantly higher (*P* = 0.025, Student's t test) in tumors with Gleason Score ≥ 8 (n= 106) than in tumors with Gleason Score ≤ 6 (n =51). Other comparisons, i.e. GS ≥ 8 vs. GS = 7, or GS = 7 vs. GS ≤ 6, did not show significant difference. *, P < 0.05.

Our previous study showed that NRIP stabilizes the AR protein and up-regulates the expression of prostate-specific antigen (PSA) [2]. Because the AR is crucial in prostate cancer progression, according to many studies [24, 28], and the PSA test is widely used for prostate cancer screening for men after the age of 50 (National Cancer Institute Website:www.cancer.gov). We were interested in evaluating the expression of NRIP in prostate cancer tissues; the immunohistochemistry results indicated that the NRIP protein was expressed in the nuclei and cytoplasm of luminal and basal cells (Figure 1A, non-neoplastic tissue). A high level of NRIP expression was defined as a score greater than or equal to 150 and a score less than 150 was defined as low expression. The result (Figure 1B) was that the percentage of cases which scored as high expression for NRIP was greater for neoplastic tissues than for non-neoplastic tissues (non-neoplastic vs. neoplastic = 57.1% vs. 81.0%; *P* < 0.005). We further divided these prostate tumors into three sub-categories (Figure 1B) according to their Gleason Scores (GS) (less than or equal to 6, equal to 7, and greater than or equal to 8). Similarly, each sub-category had a significantly greater (*P* < 0.05) percentage of high-NRIP neoplastic tissues than the non-neoplastic group (Figure 1B); indicating that high score GS patients have more NRIP expression than non-neoplastic prostate. Moreover, when the average expression scores of different sub-categories were compared, NRIP expression in the GS ≥ 8 subcategory was significantly higher than the ≤ 6 subcategory (P = 0.025) and the non-neoplastic prostate (P = 0.023) (Figure 1C). But there were no significant differences between any other pairs of these four sub-categories in terms of the percentage of high NRIP expression (Figure 1C). Collectively, NRIP expression is up-regulated in human prostate cancer and may be positively correlated with tumorigenesis; cancers with higher Gleason scores have higher levels of NRIP expression than cancer tissues with lower Gleason Scores.

### Expression of the AR protein in human prostate cancer

**Figure 2:**
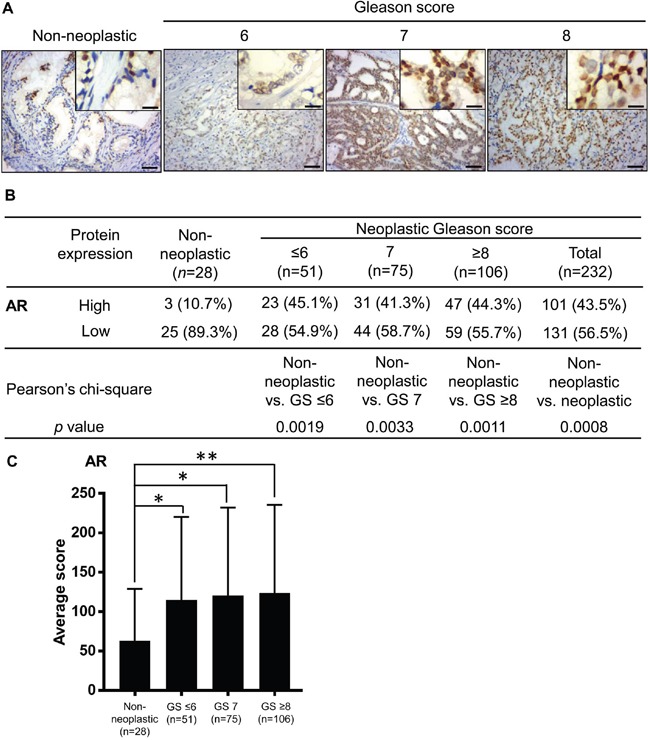
AR expression is increased in human prostate cancer tissues compared to non-neoplastic prostate tissues **A**. Representative images of immunohistochemistry staining for AR expression. Left to right represent non-neoplastic, Gleason Score ≤ 6, Gleason Score= 7, and Gleason Score ≥ 8 groups. Brown: AR. Blue: hematoxylin counterstain. Upper right insets: magnified figures, Scale bar = 12.5 μm for insets, 50 μm for others. **B**. Comparison of the AR expression levels in non-neoplastic tissues and tumors of different grades. **C**. Comparison of AR expression level, based on its average immunohistochemistry score. The calculation of the immunohistochemistry score of AR expression and definition of “high” and “low” expression was the same as in Figure 1. *, P < 0.05; **, P < 0.01.

According to many studies, the AR is important in prostate cancer progression [24, 25, 28]. However, whether AR expression is related to the prostate cancer stage and outcome remains controversial, because inconsistent results have been reported [29]. Therefore, we sought to evaluate the AR expression levels in our prostate tissues and to correlate these with tumor grading and NRIP expression. Expression of the AR was predominantly in the nucleus of non-neoplastic and neoplastic tissues (Figure 2A). Similar to NRIP, the percentage of high-AR cases was significantly greater (*P* < 0.005 for each comparison in Figure 2B) in human prostate cancer (either all tumors or any sub-category with GS ≤ 6, 7, or ≥ 8) than in non-neoplastic prostate tissues (Figure 2B). An analysis comparing the average expression scores among different sub-categories also yielded similar results (Figure 2C). Collectively, AR expression is significantly higher in our prostate cancer tissues than non-neoplastic tissues. Previously, we reported that NRIP could increase and stabilize AR protein in LNCaP cells [2]. Thereof, we further compared the expression levels of NRIP and AR in prostate cancer tissues analyzed by immunohistochemistry. Table 1 showed a significantly positive correlation (the odds ratio = 2.10, 95% confidence interval =1.04 - 4.26, *P* value = 0.0376) between the expression levels of NRIP and AR, supporting the hypothesis in our previous study that NRIP increases the expression of AR protein [2].

**Table 1:** The numbers of prostate cancer tissues with either high or low expression levels of AR and NRIP in immunohistochemistry

		NRIP			P value
		High	Low	Total	
**AR**	High	88	13	101	
	Low	100	31	131	
	Total	188	44	232	< 0.05

The staining scores (described in Methods) higher than 150 were defined as having “high” expression and otherwise as “low” expression. Odds ratio: 2.10; 95% confidence interval: 1.04 - 4.26; Chi square P value = 0.0376.

### Expression of the DDB2 protein in human prostate cancer

**Figure 3:**
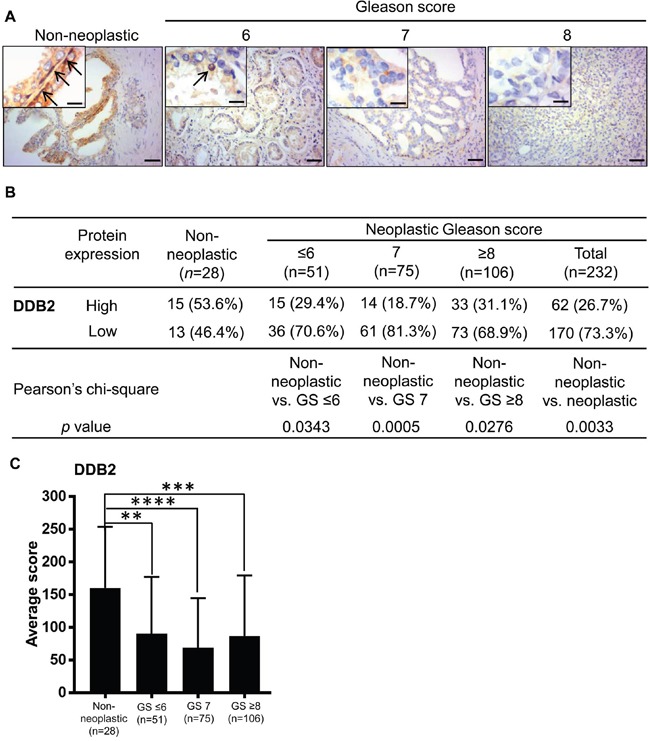
DDB2 expression is decreased in human prostate cancer tissues compared to non-neoplastic prostate tissues **A**. Representative images of immunohistochemistry staining for DDB2 expression. Brown: DDB2. Blue: hematoxylin counterstain. Upper right insets: magnified figures, Scale bar = 12.5 μm for insets, 50 μm for others. Arrow: nuclear DDB2 expression. **B**. Comparison of the DDB2 expression levels between non-neoplastic tissues and tumors of different grades. The immunohistochemistry score was calculated as described in Figure 1: However, due to the generally lower expression of DDB2 compared to NRIP and AR, the DDB2 staining scores equal to or greater than 125 was defined as “high” expression and otherwise as “low” expression. GS, Gleason score. **C**. Comparison of DDB2 expression level, based on its average immunohistochemistry score. **, P < 0.01; ***, P < 0.001; ****, P < 0.0001.

Previously, we found that DDB2 interacts with the AR [23]. In addition, DDB2 reportedly acts as a tumor suppressor in a wide range of cancers [16–21, 30, 31]. The relationship between DDB2 and prostate cancer remains unclear. We then investigated DDB2 protein expression in human prostate cancer tissues and found that the DDB2 protein was expressed predominately in the nuclei of basal cells and rarely in luminal cells in normal prostate tissues (Figure 3A), a result consistent with previous reports that DDB2 is a nuclear protein, and the nuclear signal of DDB2 expression is exclusively used for immunohistochemistry evaluation in human colon cancer tissues [18, 32]. Using a similar scoring system, we compared DDB2 expression in non-neoplastic and neoplastic tissues and found, in contrast to NRIP and AR, a greater percentage (*P* < 0.05 for each comparison in Figure 3B) of high-DDB2 cases in the non-neoplastic tissues than prostate cancers (either all tumors or any sub-category with GS ≤ 6, 7, or ≥ 8) (Figure 3B). In addition, an analysis comparing the average expression scores among different sub-categories also yielded similar results (Figure 3C). Collectively, DDB2 expression is down-regulated in human prostate cancer.

Because DDB2 reportedly promotes the ubiquitination and degradation of AR in LNCaP cell lines [23], we next examined the relationship between DDB2 and AR protein expression in human prostate cancer tissues. However, the result showed a positive correlation (odds ratio: 3.24, confidence interval: 1.77-5.96) between the AR and DDB2 in human prostate cancer tissues (Supplementary Table 1A); it contradicted *in vitro* conclusion of DDB2-degrading AR. Furthermore, by separating the original Supplementary Table 1A into two groups: one with low expression of NRIP (Supplementary Table 1B) and the other with high expression of NRIP (Supplementary Table 1C), we found that a positive correlation between the expression of DDB2 and AR (*P* = 0.00073) only existed in the group with high expression of NRIP; but not in the low-NRIP group. Since tumors are very heterogeneous; it is probable that in some tumors, high expression of AR may stimulate the expression of DNA repair-related genes including DDB2 and in these cases tumor cells may have developed other strategies to antagonize DDB2. We reasoned that a high level of NRIP might antagonize the destabilizing effect of DDB2 and protect AR; allowing tumors to express a high level of DDB2 for other purposes, a phenomenon that complies with our hypothesis.

### NRIP competes with DDB2 to protect the AR from protein degradation

**Figure 4:**
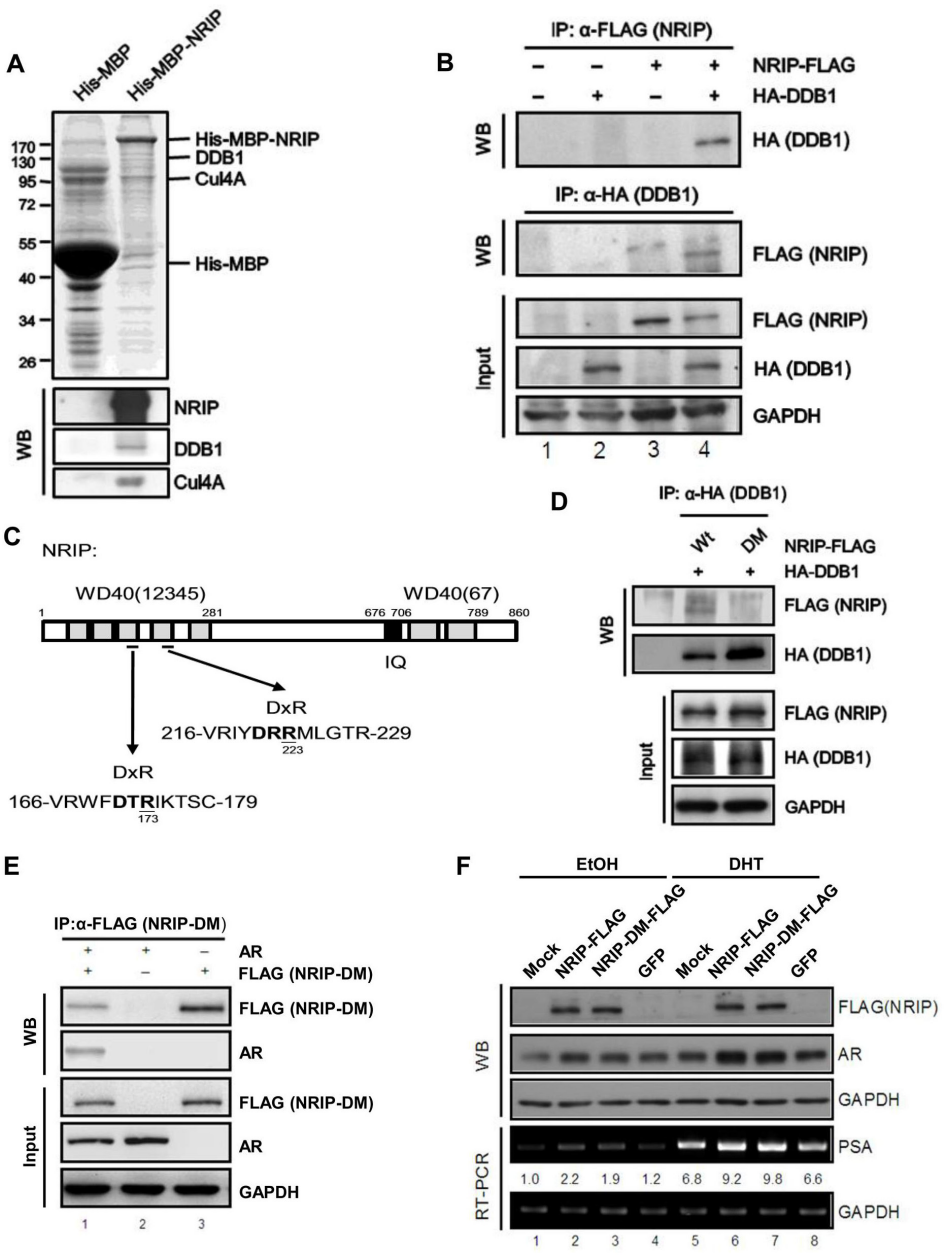
NRIP is involved in the CUL-DDB1 E3 ligase mechanism by interacting with DDB1 and associating with the DDB1-CUL4 complex through its DxR motif **A**. NRIP is involved in the CUL4A-DDB1 complex. His-MBP and His-MBP-NRIP were expressed from bacteria and purified using Ni-NTA beads. The beads conjugated with recombinant proteins were incubated with the lysates of HeLa cells and the co-purified proteins were separated by SDS-PAGE and analyzed by Coomassie blue staining (upper panel) or western blotting (lower panel) using the antibodies indicated. **B**. The interaction between NRIP and DDB1. Calcium phosphate was used to transfect 293T cells with NRIP-FLAG and HA-DDB1 plasmids. Cell lysates were collected 48 h after transfection and immunoprecipitated with anti-FLAG or anti-HA antibodies for detection of NRIP or DDB1, respectively. **C**. A schematic depiction of the protein DxR motif of NRIP. Aspartic acids at 173 and 223 in the regions bounded by amino acid residues 166 to 179 and 216 to 229 were replaced by alanine using site-directed mutagenesis and named NRIP-DM. **D**. NRIP-DM lost DDB1 binding. 293T cells were cotransfected with the wild-type NRIP or NRIP-DM mutant with HA-DDB1, cell lysates were extracted and immunoprecipitated with anti-HA antibodies for detection of DDB1 and immunoblotted with anti-FLAG for detection of NRIP and NRIP-DM. **E**. NRIP-DM interacted with AR. After cotransfection of 293T cells with FLAG-tagged NRIP-DM and AR, cell extracts were subjected to immunoprecipitation with anti-FLAG or anti-AR antibodies and the immunoprecipitated proteins analyzed by western blotting using anti-AR or anti-FLAG antibodies, reciprocally. The loading of the cell extracts represents 10% of the input used for immunoprecipitation to assess comparable protein levels. **F**. AR protein stabilization was independent of NRIP and DDB1 interaction. LNCap cells were transfected with NRIP-FLAG, NRIP-DM-FLAG, and GFP, respectively. After 24 h, cells were treated with 10 nM DHT for 24 h. Proteins and RNAs were extracted by RIPA and Trizol reagent, respectively. The protein expression of NRIP and AR was detected by western blot analysis with anti-FLAG and anti-AR primary antibodies. The expression of PSA was detected by RT-PCR. GAPDH was used as a loading control.

NRIP is a DCAF protein and is also known as DCAF6 and IQWD1 [3]. DCAFs are a family of proteins that associate with DDB1 and CUL4 and serve as receptors for substrates that recruit E3 ligase to the DDB1-CUL4 complex [33]. Because NRIP expression is correlated with AR expression in human prostate tumors (Table 1); we were interested in understanding the mechanism for positive correlation between NRIP and AR. Therefore, we examined the interactions of NRIP with proteins pulled down from nuclear extracts of HeLa cells. Based on molecular weight of CUL4A (87 kD) and DDB1 (127 kD), we localized the positions of these two proteins in gel (Figure 4A, upper panel); and western blot assay confirmed each relative location of CUL4A and DDB1 (lower). Interestingly, the western blot results suggested that much more CUL4A molecules (strong band) than DDB1 bound to NRIP; one reason could be the CUL4A-DDB1 complex containing more CUL4A molecules than DDB1, the other might be stronger anti-CUL4A antibody than anti-DDB1 antibody. Hence, Figure 4A indicated that NRIP might bind to the DDB1-CUL4A complex. To determine whether NRIP binds to DDB1 *in* cells, the interaction between NRIP and DDB1 was examined *in* 293T cell and observed reciprocally (Figure 4B, lane 4). Recent reports have shown that DCAF proteins contain WD40 repeats and the interaction between DCAFs and DDB1 is through a WDXR motif in the WD40 repeat [3, 34]. The WDXR motif in WD40 repeats is followed occasionally by an X-Arg dipeptide or X-Lys dipeptide. As shown in Figure 4C, NRIP contains two predicted WDXR motifs in its third and fourth WD40 repeat, based on the amino acid analysis. To further map the DDB1-interaction site of NRIP, we generated a double point mutant (NRIP-DM with arginine [R] at aa 173 and aa 223 changed by site-directed mutagenesis to alanine [A]) in these two WDXR motifs. To confirm that the two WDXR motifs on NRIP bind DDB1, 293T cells were cotransfected with the expression vectors for HA-tagged DDB1 and FLAG-tagged NRIP wild type or NRIP-DM and co-immunoprecipitation was performed, showing that NRIP-DM failed to bind to DDB1 (Figure 4D, lane 2). Hence, these two WDXR motifs of NRIP are necessary for DDB1 binding. Previously we showed that NRIP binds with the AR and stabilizes it [2]. We sought to determine whether loss of DDB1 binding affects AR protein stability and found that NRIP-DM associated with AR reciprocally in cells (Figure 4E, lane 1). NRIP-DM can bind to AR but not to DDB1. We overexpressed NRIP and NRIP-DM in LNCaP cells with or without DHT treatment to observe the protein expression and transcriptional activity of AR by western blotting (WB) and RT-PCR analysis of PSA. The WB showed that both overexpression of NRIP and NRIP-DM enhanced the expression of AR protein compared to GFP and mock control with (Figure 4F, lanes 6 and 7). The transcription of PSA, a target gene of AR, was upregulated in both NRIP and NRIP-DM overexpressing LNCaP cells, with DHT treatment (Figure 4F). There was slightly increased AR protein by NRIP-DM mutant in comparison with GFP alone in the absence of DHT (Figure 4F lane 3 and lane 4); the mild increase of AR was coupled with the mild increase of PSA RNA from three individual experiments. In summary, both NRIP and NRIP-DM (DDB1-binding deficient NRIP) can enhance AR protein stability.

**Figure 5:**
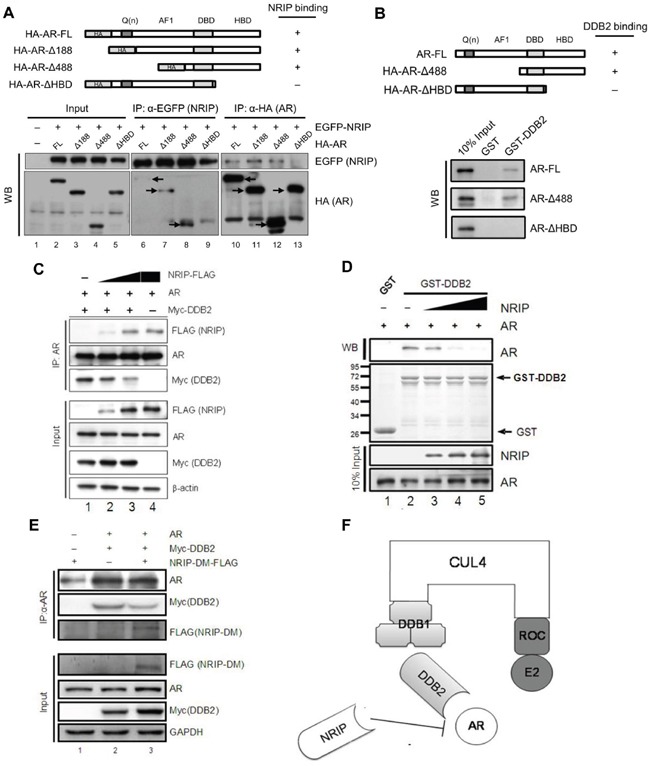
NRIP competes with DDB2 for AR but not DDB1 binding **A**. NRIP binds the AR at HBD. Upper panel: a schematic figure of HA-AR-FL and various mutants of HA-AR, including HA-ARΔ188, HA-ARΔ488 and HA-ARΔHBD. This figure indicates that the C-terminal HBD of the AR was required for NRIP binding. Lower panel: 293T cells were cotransfected with EGFP-NRIP and various mutant forms of the AR, as shown in the upper panel. After 24 h, proteins were extracted by RIPA buffer and subjected to immunoprecipitation with anti-EGFP or anti-HA antibodies. The expression of EGFP-NRIP, HA-AR-FL and HA-AR mutants was detected by western blotting with anti-EGFP and anti-HA antibodies, respectively. Arrows: the positive signals detected by anti-HA antibody. Lanes 1 to 5: 10% of the input used for immunoprecipitation to assess comparable protein levels. Lanes 6 to 9: the immunoprecipitation performed with anti-EGFP. Lanes 10 to 13: the immunoprecipitation performed with anti-HA. **B**. The C-terminal HBD of the AR is required for DDB2 binding. Upper panel: a schema of HA-AR-FL and various mutants of HA-AR, including HA-ARΔ488 and HA-ARΔHBD. This figure indicates that the C-terminal HBD of the AR was required for DDB2 binding. Lower panel: the HA-AR-FL, HA-ARΔ488 and HA-ARΔHBD were incubated with GST-DDB2 and followed by GST-pull down assay. The binding of HA-AR-FL, HA-ARΔ488 and HA-ARΔ with DDB2 were then analyzed by western blotting. **C**. NRIP competes with DDB2 for AR interaction. Cotransfection of 293T cells with expression vectors, including AR and Myc-DDB2, along with increasing amounts of FLAG-NRIP, was performed as indicated. The empty expression vector was used as a filler to maintain a constant amount of total plasmid DNA in all transfections. The cell lysates were subjected to immunoprecipitation 48 h after transfection using anti-AR antibodies and then to western blotting using anti-FLAG and anti-Myc antibodies to detect NRIP and DDB2, respectively. **D**. *In vitro* competition of the binding of DDB2 and the AR in the presence of NRIP. GST-DDB2 produced from bacteria was incubated with AR protein and various amounts of NRIP, followed by *in vitro* GST-pull down assay. After adding NRIP, the interaction of AR and DDB2 decreased. **E**. NRIP-DM mutant competes with DDB2 for binding AR in cells. To determine whether AR or DDB1 binding is required for NRIP displacement of DDB2, 293T cells were transiently transfected with two or all three of the following: FLAG-tagged NRIP-DM, Myc-DDB2, and HA-AR. The total amount of plasmid DNA was kept constant by adding empty plasmid DNA. The cell extracts were harvested after 48 h and subjected to immunoprecipitation with AR antibody. The immunoprecipitates were analyzed by western blotting for the detection of the AR, Myc-DDB2, and FLAG-NRIP-DM with the antibodies indicated. The inputs represent 5% of the cell extracts used in immunoprecipitation. **F**. A schematic model of AR degradation via DDB2-DDB1-CUL4A-ROC1. NRIP also interacts with CUL4A-DDB1 but functions to stabilize AR protein. The schema shows that NRIP competed with DDB2 for binding to DDB1 or AR.

Previously, DDB2 was found to degrade the AR via the CUL4A-DDB1 E3 ligase complex in LNCaP cells [23]. Here, we demonstrated that DDB2 expression is reduced in human prostate cancer (Figure 3). On the other hand, NRIP, like DDB2, is a DCAF-associated protein and can form a CUL4A-DDB1 complex (Figure 4A) and NRIP can protect AR protein stability. To determine whether NRIP prevents DDB2-mediated degradation of the AR, we first identified the domain of the AR that binds NRIP. The structure of the AR comprises an N-terminal regulatory domain, including activation function 1 (AF-1), a DNA binding domain (DBD), and a C-terminal hormone-binding domain (HBD) [35]. Reciprocal coimmunoprecipitation indicated that AR-ΔHBD could not interact with NRIP by NRIP pull-down assay (Figure 5A, lower panel, lane 9); similarly, no NRIP band was observed by AR-ΔHBD pull-down assay (lane 13). Collectively, NRIP bound to AR through its hormone-binding domain. From NRIP pull-down assay, truncated AR-Δ188 and AR-Δ488 mutants consistently had stronger binding affinity to NRIP than AR wild type from three individual experiments, indicating that the structures of these truncated AR proteins might be more feasibly to interact with NRIP. Taken together, NRIP binds to the HBD of the AR.

On the other hand, DDB2 also can bind to the AR [23]. To map which domain of AR DDB2 binds to, we used a GST-pull down assay to investigate DDB2 binding to various AR mutants. As shown in Figure 5B, the binding of DDB2 to the AR was abrogated when the C-terminal HBD was deleted from the AR (Figure 5B, lower panel). The data in Figure 5A and 5B indicate that NRIP and DDB2 bind to the same domain (HBD) of the AR protein. Therefore, it was interesting to investigate whether NRIP can interfere with the association between DDB2 and the AR; LNCaP cells were cotransfected with plasmids encoding Myc-DDB2 and increasing amounts of NRIP-FLAG. Coimmunoprecipitation with anti-AR antibodies (Figure 5C) showed that less DDB2 protein was associated with the AR protein in the presence of a high dose NRIP (Figure 5C, lane 3). *In vitro* competition by GST-pull down assay also showed that increased NRIP protein could attenuate GST-DDB2 binding to the AR protein (Figure 5D, lanes 3 to 5). These findings indicate that NRIP inhibits the association between DDB2 and AR. Since NRIP binds either DDB1 or AR, in order to distinguish whether NRIP's protection of AR from DDB2 degradation is through NRIP associating with DDB1-CUL4A complex or binding to AR; we chose to use NRIP-DM mutant that lacks DDB1 binding but still interacts with AR for competition assay. LNCaP cells were cotransfected with an expression vector for Myc-tagged DDB2 alone or with an expression vector for FLAG-tagged NRIP-DM. Co-immunoprecipitation indicated that overexpression of NRIP-DM still interfered with the association between DDB2 and the AR (Figure 5E, lane 3). Taken together, the evidence suggests that the effect of NRIP on AR expression does not depend on its association with the DDB1-CUL4 complex and that displacement of DDB2 by NRIP will result in interaction with the AR protein and, thereby, in AR protein stabilization (Figure 5F).

### Co-expression of NRIP and the AR are found frequently in low-DDB2 expressing cribriform prostate cancer

**Figure 6:**
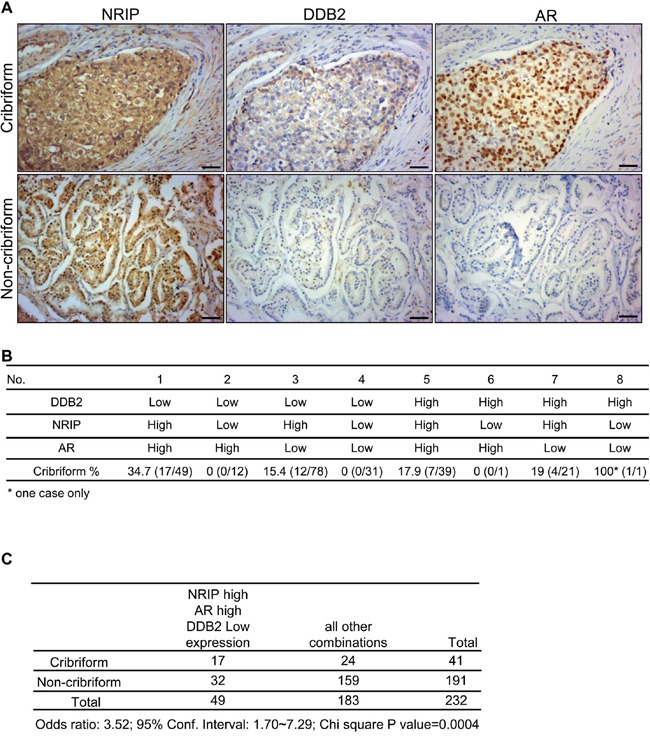
The expression pattern with high levels of NRIP and AR, together with a low level of DDB2, is more frequently found in human cribriform prostate cancer tissues **A**. Representative immunohistochemistry staining images of NRIP, DDB2 and AR expression in non-cribriform and cribriform prostate cancer tissues. Upper panel: cribriform tumors. Lower panel: non-cribriform tumors. Brown: the positive stain of NRIP, DDB2 or AR. Blue: hematoxylin counterstain. Scale bars: 50μm. **B**.. Distribution percentage of eight different combinations of expression pattern of NRIP, DDB2 and AR in non-cribriform and cribriform prostate cancer tissues. Percentage = cribriform /cribriform + non-cribriform × 100%. **C**. A chi square test to compare the distribution of NRIP-high/AR-high/DDB2-low expression pattern and all other combinations of expression pattern for NRIP, DDB2 and AR in non-cribriform and cribriform prostate cancer tissues.

In the prostate cancer cell line LNCaP, NRIP displaced DDB2 to prevent DDB2 degrading the AR in the CUL4-DDB2-E3 ligase complex (Figure 5). We sought to determine whether, in human prostate cancer tissues, the expression patterns of these three proteins could match the specific mode of NRIP-DDB2-AR interaction revealed by our *in vitro* study. Most prostate cancer tissues are acinar adenocarcinomas [36] and can be further categorized into several subtypes, based on their cytoarchitectural features [37–39]. Among them, cancer with the cribriform pattern, or the intraductal carcinoma of the prostate [37–39], is a special subtype of prostate cancers associated with poor prognosis. It is named after the feature of solid lesions comprising tumor cells that span or fill glandular lumens but preserve, at least focally, a basal cell lining. Initially we noticed that ∼ one fifths of our prostate cancer samples were cribriform tumors; we then analyzed the expression patterns of NRIP, AR, and DDB2 in the context of the prostate cancers with or without the cribriform pattern (Figure 6A and 6B). As shown in Figure 6B, the highest percentage of tumors (34.7%) with cribriform pattern was found in the group that were low for DDB2, high for NRIP, and high for AR. This is consistent with our hypothesis model *in vitro* (Figure 5F). Furthermore, high expression levels of both NRIP and AR, along with low expression levels of DDB2, compared with all the other seven possible expression patterns of these three proteins (Figure 6C), were found more frequently (odds ratio: 3.52, 95% confidence interval: 1.70-7.29, *P* < 0.0005) in the same lesions of prostate cancer with the cribriform pattern than in all other types prostate cancer, which were categorized as non-cribriform tumors. On the other hand, because the majority (73%; 170/232) of our prostate cancer tissues expressed low levels of DDB2 (Supplementary Table 2), when only those low-level DDB2 tumors were considered in comparison, it became more obvious that high expression levels of both NRIP and AR were more frequently detected in the cribriform tumors (odds ratio: 4.83, 95% confidence interval: 2.09-11.15, P < 0.005), while other combinations of expression were found more frequently in non-cribriform tumors (Supplementary Table 2). In addition, we found that high expression levels of NRIP, AR or both were detected more frequently in the cribriform tumors than non-cribriform tumors (Supplementary Table 3). Collectively, the specific expression pattern of NRIP, AR and DDB2 in the cribriform type of human prostate cancer is consistent with our hypothesis that NRIP protects against AR degradation by DDB2 in the CUL4-DDB1 E3 ligase complex, in at least a subset of prostate cancers.

## DISCUSSION

In this study, we found that either NRIP (Figure 1) or the AR (Figure 2) was highly expressed in human prostate cancer tissues. Consistently, there was a positive correlation between the expression of NRIP and the AR (Table 1). The roles of the AR in prostate cancer are complex; the AR can be either tumor-inductive or tumor-suppressive, depending on which histological regions cancer cells are derived from [40, 41]. For example, when overexpressed in cancers derived from basal epithelial cells, the AR functions as a tumor suppressor to inhibit the proliferation of basal cells and drive them into differentiation, resulting in the inhibition of prostate cancer metastasis [40, 42]. In contrast, the AR is a survival factor that promotes the proliferation of luminal cellsthat constitute more than 99% of prostate tumor epithelial cells [40, 43]. Here, we report that AR expression increased in prostate cancer tissues and support the proposed model of the AR as an oncogene in cancer. Like the AR, NRIP is an androgen-regulated gene [2] and our current data support the view that it acts as an oncoprotein in prostate cancer. In this regard, there are quite a few precedents, such as prostatic secretory protein-94, PSA, and TMPRSS2, all of which are products of androgen-regulated genes and enhance the progression of prostate cancer [44]. In this study, we reveal NRIP to be a new member of this list of proteins implicated in the pathogenesis of prostate cancer.

Several studies have shown that DDB2 acts a role as a tumor suppressor in a wide range of cancers, including UV-induced skin cancers [13], ovarian cancer [17], breast cancer [19, 20] and colon cancer [18]. Consistently, our data support that DDB2 functions as a tumor suppressor in prostate cancers. More importantly, our previous *in vitro* study indicated that DDB2 enhances the ubiquitination and proteasomal degradation of the AR, mediated by the DDB2-DDB1-Cul4 complex [23]. However, when the correlation between the expression levels of DDB2 and the AR in human prostate cancer tissues was analyzed, a positive correlation of the expression of these two proteins was revealed (Supplementary Table 1A), an observation that may not match our proposed model of DDB2-mediated AR degradation in the LNCaP cell line. However, this phenomenon may be explained by the fact that DDB2, in addition to its role as a tumor suppressor in many cancers, as outlined above, DDB2 also acts as damaged DNA binding protein to detect cancer chromosome instability [45]. There is evidence that DNA repair-related genes are activated by the AR in LNCaP cells treated with androgen [46]. Accordingly, DDB2 expression may be induced in prostate cancer tissues with high levels of AR expression. Interestingly, in another hormone-related cancer, breast cancer, overexpression of DDB2 has been reported in tumors of the non-invasive type [20] and another study demonstrated that the expression of DDB2 is higher in ER-positive than ER-negative breast tumors [47]. These studies also revealed that high levels of DDB2 expression in cancer tissues might be involved in DNA repair. Therefore, the correlation of AR and DBB2 expression in prostate cancers seems logical, because high levels of AR expression in cancer cells may activate the DNA repair mechanism that up-regulates DDB2 expression.

DCAFs are DDB1- and CUL4-binding proteins that are part of a large family of WD40 repeat-containing proteins [33, 48–50]. Almost all of these DCAFs have two conserved DxR motifs within the WD40 domain that are essential for DDB1 binding. DDB2 is the best-characterized DCAF and is needed for DDB1–CUL4A-mediated ubiquitination of certain substrates [8, 48]. Like DDB2, NRIP contains a double DxR box (Figure 4C), which is predicted to be solvent-exposed and located at the bottom of the WD40 propeller fold (an important determinant for the binding to DDB1). In the present study, we demonstrated that NRIP, like DDB2, interacts with the DDB1-CUL4A complex (Figure 4). Furthermore, we found that NRIP competes with DDB2 for binding sites on the DDB1-CUL4A complex, because these two DCAFs can both interact with DDB1 and AR to form the DDB1-CUL4A complex and share the same hormone-binding domain (HBD) of AR for interaction (Figure 5A and 5B). It is possible that NRIP blocks interaction with either DDB1-DDB2 or DDB2-AR. Our data showed that both NRIP and NRIP-DM (with an AR binding site but no DDB1 binding site) interfered with the interaction of DDB2 and AR, indicating that NRIP actually competes with DDB2 for AR binding but has no effect on DDB1 (Figure 5E). In a previous study [23], we showed that, in the absence of DDB1, CUL4A in LNCaP cells increases the AR protein level but introduction of DDB1 reduces AR protein, implying that once NRIP complexes with DDB1-CUL4A, the concentration of DDB1 and CUL4A needed to assemble into the AR-degrading DDB2-DDB1-CUL4A complex is too low in certain compartments. These data show that NRIP prevents AR degradation by interfering with DDB2 assembly into DDB2-DDB1-CUL4A complexes. On the other hand, NRIP-DM mutant can interact with AR but not DDB1 and can stabilize AR as well as wild-type NRIP. Collectively, there are at least two mechanisms for NRIP to stabilize AR; one is through directly association with AR; the other is through forming a complex with DDB1-CUL4A to decrease its binding to DDB2. Additionally, as for the distribution of these proteins in normal prostate tissues, NRIP was expressed both in basal cells and luminal cells; AR mostly in luminal cells and few in basal cells; DDB2 mostly in basal cells and rarely in luminal cell. It will be interesting for future to decide how and where for NRIP and DDB2 to compete either CUL4A-DDB1 complex or AR in normal condition.

There are two reasons why we separated tumors into cribriform and non-cribriform subtypes in our analysis. First, in our initial assessment of pathology slides of prostate tumors that were immuno-stained for DDB2, NRIP, and AR, we already found that some cribriform tumors had distinct expression patterns for these 3 proteins and thus we decided to clarify it using complete statistical analysis. Second, prostate cancers with the cribriform pattern have been well recognized as high-Gleason score tumors [37–39, 51]. The presence of the cribriform pattern is a strong predictor of metastasis, as well as disease-specific death, in patients treated with radical prostatectomy [52]. A study of 241 radical prostatectomy specimens with the highest Gleason grade of 4 found that patients with tumors with the cribriform pattern tend to have biochemical recurrence and metastasis after radical prostatectomy [53]. It also has been reported that cribriform cancer carries a distinctly adverse clinical outcome [53, 54]. Our results indicated that a specific pattern of high expression of NRIP and AR and simultaneous low expression of DDB2 was detected more frequently in cribriform tumors than in non-cribriform tumors (Figure 6). Such a unique expression combination is reasonable from the perspective of our hypothetical model of NRIP's role in protecting AR degradation from the DDB2-DDB1-CUL4A. In addition, cribriform prostate cancer possesses other unique molecular and genetic features; for example, p63, high molecular weight cytokeratin, and TMPRSS2-ERG gene fusion are reportedly significantly more common in cribriform tumors than non-cribriform tumors [55, 56]. The cross talks between these abnormally expressed molecules and the NRIP/DDB2-AR system may be related to the development of the specific morphological and clinical features seen in cribriform tumors and deserves further study.

In summary, we propose that NRIP and DDB2, through competitive binding to the AR, antagonize each other's function in maintaining AR homeostasis and disruption of the balance between these two proteins may contribute to the pathogenesis of a specific type of aggressive prostate cancer.

## MATERIALS AND METHODS

### Human prostate cancer

The human prostate biopsies were obtained from the National Taiwan University Hospital (NTUH) Tissue Bank; prostate cancer tissue arrays (serial numbers: PR953, PR955, PR483b, PR753 and PR8010) were purchased from US Biomax, Inc (Rockville, MD, USA). Studies involving human prostate tissues were approved by the Institutional Review Boards at National Taiwan University (NTU). The Gleason scores of each sample were provided by the original sources, NTUH Tissue Bank and US Biomax Inc.

### Immunohistochemistry

For immunohistochemistry staining, the paraffin-embedded sections were probed with anti-NRIP (GeneTex, GTX10595; 1:100), DDB2 (Santa Cruz, sc-25368; 1:50), and AR (DAKO, M356201; 1:100) antibodies. All these antibodies have been published previously in the literature for immunohistochemistry purpose [57, 58]. The specificity of each antibody was confirmed in human prostate cancer tissue by immunohistochemistry without each primary antibody.

### Immunohistochemistry scores of protein expressions

Immunohistochemistry scores of DDB2, NRIP and AR expression were obtained by multiplying the intensity scores and the extent scores, which were given double-blindly by two different researchers with pathology training. The intensity score was from zero to three, with 0 representing no staining (negative), 1 representing weak staining, 2 representing intermediate staining, while 3 representing strong staining [59]. The extent score was calculated by the percentage (scored as 0-100%) of entire prostate gland areas on the slides of patient specimens from NTUH or of each dot on the tissue arrays from US Biomax Inc. The total score was obtained by multiplication of intensity by extent, from 0 to 300. The tissues scoring higher than 150 were defined as having high expression for NRIP and AR staining. As for DDB2, the cut-off value of high expression was set at 125 [58]. A recent study comparing the results between the automated measures (such as ImageJ) and manual scoring concluded that both methods resulted in essentially identical scores when applied to patient biopsies [60].

### Reverse-transcriptase polymerase chain reaction (RT-PCR)

Total RNAs were isolated with TRIzol reagent (Invitrogen) and treated with DNase (RQ1, Promega) to remove genomic DNA according to the manufacturers’ instructions. One microgram of RNA was reverse-transcribed into cDNA by SuperScript™ III First-Strand Synthesis System (Invitrogen) and subjected to PCR. The forward primer sequence of PSA was 5′-ATGTGGGTCCCGGTTGTCTTCCTCACC-3′. The reverse primer sequence of PSA was 5′-TCAGGGGTTGGCCACGATGGTGTCCTT-3′. The forward primer sequence of GAPDH was 5′-ACCTTCAACACCCCAGCCATG-3′. The reverse primer sequence of GAPDH was 5′-CTG GAAGAGTGCCTCAGGGCA-3′.

### Plasmid construction

The construction of FLAG-NRIP and His-NRIP were described previously [4]. The FLAG-NRIP-double mutant (DM, the DxR mutations in which the arginine [R] residues at aa 173 and aa 223 were changed to alanine [A] with the intact AR binding site but without functional DDB1 binding site) plasmid were mutated and generated from FLAG-NRIP in such a way that the aspartic acids at 173 and 223 in the regions bounded by amino acid residues 166 to 179 and 216 to 229 were replaced by alanine using PCR-based site-directed mutagenesis (Figure 4C). The construction of GST-DDB2 was described previously [23]. The construction of plasmids-pcDNA3.0-AR was described previously [2]. Plasmids expressing HA-DDB1, Myc-DDB2 were kindly provided by Dr. Yun Xiong (University of North Carolina, Chapel Hill, NC), the pSG5-AR or deletion mutants were from Dr. Andrew C.B. Cato (Forschungszentrum Karlsruhe, Institute of Genetics, Karlsruhe, Germany) [61].

### Cell culture and transfection

293T cells were transfected with the plasmids indicated in growth medium using calcium phosphate [2, 4, 23]. LNCaP cells were transfected with FuGENE 6 (Roche Applied Science) or SuperFect (Qiagen) transfection reagents according to the manufacturers' instructions [1].

### Coimmunoprecipitation and western blotting

Proteins were extracted from transfected 293T and LNCap cells and subjected to coimmunoprecipitation assay [2, 23] and western blot analysis for primary antibody incubation. Antibodies included anti-HA (1:2000, Abcam, MA, USA), anti-Flag (1:2000, Sigma, MO, USA), anti-AR (1:1000, Santa Cruz, TX, USA), anti-NRIP (1:1000, Abnova, Taipei, Taiwan) and anti-GAPDH (1:5000, Abfronteier, Seoul, Korea).

### Statistical analysis

Chi-square (χ^2^) test or Student's t-test were used to evaluate the differences between two different parameters (e.g. protein expression scores and Gleason scores). A P-value of less than 0.05 was considered statistically significant.

## SUPPLEMENTARY MATERIALS TABLES


