# Supplementary material for: NRIP/DCAF6 stabilizes the androgen receptor protein by displacing DDB2 from the CUL4A-DDB1 E3 ligase complex in prostate cancer
Source: Oncotarget. 2017 Feb 14;8(13):21501–15. doi: 10.18632/oncotarget.15308 (PMC5400601; doi:10.18632/oncotarget.15308)
Supplement: Supplementary file 1 [file oncotarget-08-21501-s001.pdf]

NRIP/DCAF6 stabilizes the androgen receptor protein by displacing DDB2 from the CUL4A-DDB1 E3 ligase complex in prostate cancer

SUPPLEMENTARY TABLES

Supplementary Table 1: Positive correlation between DDB2 and AR expression in prostate cancer patients

A

		DDB2		
		**High**	**Low**	**Total**
**AR**	High	40	61	101
	Low	22	109	131
	Total	62	170	232

Odds ratio: 3.24; 95% Conf. Interval: 1.77~5.96; Chi square P value=0.00009914.

B

Low NRIP

		DDB2		
		**High**	**Low**	**Total**
**AR**	High	1	12	13
	Low	1	31	32
	Total	2	43	45

Odds ratio: 2.58; 95% Conf. Interval: 0.15~44.70; Chi square P value=0.500; Fisher's P=0.499.

C

High NRIP

		DDB2		
		**High**	**Low**	**Total**
**AR**	High	39	49	88
	Low	21	78	99
	Total	60	127	187

Odds ratio: 2.96; 95% Conf. Interval: 1.56~5.60; Chi square P value=0.00072893.

Supplementary Table 2: The NRIP-high/AR-high expression pattern, compared to all three other combinations of NRIP and AR expression, is more frequently detected in DDB2-low cribriform prostate cancer tissues than non-cribriform cancers

	DDB2 Low expression		
	Both NRIP and AR: high	NRIP & AR: all other combinations	Total
**Cribriform**	17	12	29
**Non-cribriform**	32	109	141
**Total**	49	121	170

Odds ratio: 4.83; 95% Conf. Interval: 2.09~11.15; Chi square P value=0.0001.

Supplementary Table 3: High levels of NRIP, AR, or both are more frequently seen in cribriform tumors

	Cribriform	Non-cribriform	Odds ratio	95% intervals	*P*
NRIP (high : low)	40 : 1	147:44	11.97	1.60 - 89.60	0.0010*
AR (high : low)	24 : 17	77:114	2.09	1.05 - 4.15	0.0327
NRIP & AR (high : others#)	24 : 17	64: 127	2.80	1.40-5.59	0.0027
DDB2 (high : low)	12 : 29	50:141	1.17	0.55-2.46	0.6892

*, by Fisher's exact test, all others: chi-square test.
#, others meaning the sum of all other three combinations of NRIP and AR levels.
